# (2*E*)-3-[4-(Benz­yloxy)phen­yl]-1-(2,6-dichloro-3-fluoro­phen­yl)prop-2-en-1-one

**DOI:** 10.1107/S1600536812046855

**Published:** 2012-11-17

**Authors:** Aletti S. Praveen, Hemmige S. Yathirajan, Thomas Gerber, Benjamin van Brecht, Richard Betz

**Affiliations:** a University of Mysore, Department of Studies in Chemistry, Manasagangotri, Mysore 570 006, India; b Nelson Mandela Metropolitan University, Summerstrand Campus, Department of Chemistry, University Way, Summerstrand, PO Box 77000, Port Elizabeth, 6031, South Africa

## Abstract

In the title compound, C_22_H_15_Cl_2_FO_2_, a chalcone derivative featuring a threefold-halogenated aromatic substituent, the conformation about the C=C bond is *E*. In the crystal C—H⋯F and C—H⋯Cl contacts connect the mol­ecules into undulating sheets parallel to (101). In addition, C—H⋯π inter­actions are also present.

## Related literature

For background to possible applications of chalcones in pharmacy and industry, see: Lin *et al.* (2002 ▶); Modzelewska *et al.* (2006 ▶); Svetaz *et al.* (2004 ▶); Sarojini *et al.* (2006 ▶). For related structures, see: Yathirajan *et al.* (2006 ▶); Betz *et al.* (2012 ▶). For graph-set analysis of hydrogen bonds, see: Etter *et al.* (1990 ▶); Bernstein *et al.* (1995 ▶).
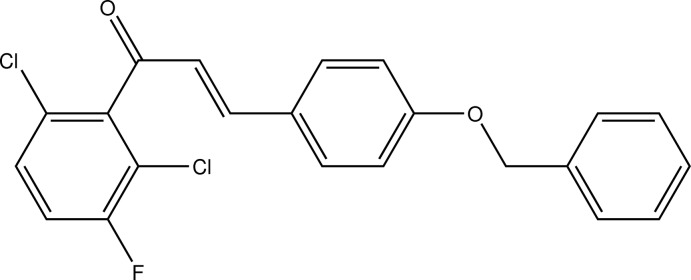


## Experimental

### 

#### Crystal data

C_22_H_15_Cl_2_FO_2_
*M* *_r_* = 401.24
Monoclinic, 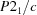

*a* = 9.1977 (2) Å
*b* = 21.6887 (4) Å
*c* = 11.6072 (2) Å
β = 124.629 (1)°
*V* = 1905.29 (6) Å^3^
*Z* = 4
Mo *K*α radiation
μ = 0.36 mm^−1^
*T* = 200 K
0.40 × 0.17 × 0.14 mm

#### Data collection

Bruker APEXII CCD diffractometer
Absorption correction: multi-scan (*SADABS*; Bruker, 2008 ▶) *T*_min_ = 0.681, *T*_max_ = 0.746
17237 measured reflections
4712 independent reflections
3729 reflections with *I* > 2σ(*I*)
*R*_int_ = 0.027

#### Refinement

*R*[*F* ^2^ > 2σ(*F* ^2^)] = 0.041
*wR*(*F* ^2^) = 0.101
*S* = 1.02
4712 reflections
244 parameters
H-atom parameters constrained
Δρ_max_ = 0.40 e Å^−3^
Δρ_min_ = −0.27 e Å^−3^

### 

Data collection: *APEX2* (Bruker, 2010 ▶); cell refinement: *SAINT* (Bruker, 2010 ▶); data reduction: *SAINT*; program(s) used to solve structure: *SHELXS97* (Sheldrick, 2008 ▶); program(s) used to refine structure: *SHELXL97* (Sheldrick, 2008 ▶); molecular graphics: *ORTEP-3* (Farrugia, 2012 ▶) and *Mercury* (Macrae *et al.*, 2008 ▶); software used to prepare material for publication: *SHELXL97* and *PLATON* (Spek, 2009 ▶).

## Supplementary Material

Crystal structure: contains datablock(s) I, global. DOI: 10.1107/S1600536812046855/nc2298sup1.cif

Supplementary material file. DOI: 10.1107/S1600536812046855/nc2298Isup2.cdx

Structure factors: contains datablock(s) I. DOI: 10.1107/S1600536812046855/nc2298Isup3.hkl

Supplementary material file. DOI: 10.1107/S1600536812046855/nc2298Isup4.cml

Additional supplementary materials:  crystallographic information; 3D view; checkCIF report

## Figures and Tables

**Table 1:** Hydrogen-bond geometry (Å, °) *Cg* is the centroid of the C21–C26 ring.

*D*—H⋯*A*	*D*—H	H⋯*A*	*D*⋯*A*	*D*—H⋯*A*
C23—H23⋯F1^i^	0.95	2.55	3.375 (2)	145
C34—H34⋯Cl1^ii^	0.95	2.80	3.5462 (18)	136
C14—H14⋯*Cg* ^iii^	0.95	2.51	3.297 (2)	140

Symmetry codes: (i) 
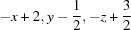
; (ii) 
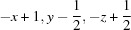
; (iii) 
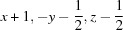
.

**Fig. 1.**
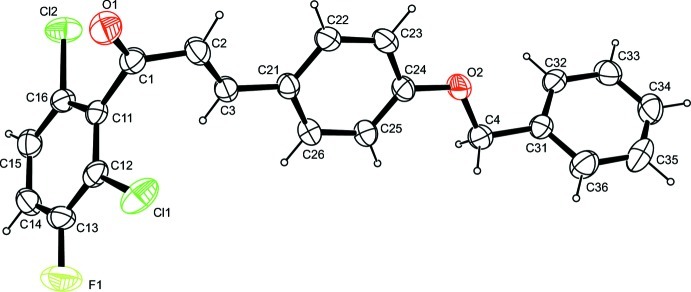
The molecular structure of the title compound, with atom labels and anisotropic displacement ellipsoids (drawn at 50% probability level).

## supplementary crystallographic information

### Comment

Chalcones are α-β-unsaturated ketones containing a reactive Michael system. Some substituted chalcones and their derivatives have been reported to possess interesting biological properties such as antitubercular (Lin *et al.*, 2002), anticancer (Modzelewska *et al.*, 2006) and antifungal (Svetaz *et al.*, 2004) activity. Chalcones also find application as organic nonlinear optical materials for their SHG conversion efficiency (Sarojini *et al.*, 2006). The crystal structures of some chalcones have been reported (Yathirajan *et al.*, 2006; Betz *et al.*, 2012). As part of our ongoing studies on chalcones, the title compound was synthesized.

The C=C bond in the Michael system is (*E*)-configured. The least-squares planes defined by the respective carbon atoms of the two terminal aromatic moieties intersect at an angle of 48.50 (10) ° and enclose angles of 62.82 (11) ° and 74.58 (8) ° with the least-squares plane defined by the carbon atoms of the central phenyl group. The larger of the latter two angles is created by the halogenated phenyl moiety (Fig 1).

In the crystal, intermolecular C–H···F and C–H···Cl contacts whose range invariably falls by more than 0.1 Å below the sum of van-der-Waals radii of the corresponding atoms are observed. These contacts are exclusively supported by hydrogen atoms on the central as well as the terminal non-halogenated phenyl group and connect the molecules to undulated sheets parallel to [1 0 1]. In addition, C–H···π interactions are present. Details about metrical parameters of these contacts as well as information about their symmetry can be found in Table 1. In terms of graph-set analysis (Etter *et al.*, 1990; Bernstein *et al.*, 1995), the C–H···F as well as the C–H···Cl contacts necessitate a *C*^1^_1_(11)*C*^1^_1_(17) descriptor on the unary level. The shortest intercentroid distance between two aromatic systems was found at 4.5552 (12) Å and is apparent between the central as well as the halogenated phenyl moiety in neighbouring molecules.

### Experimental

To a stirred solution of 1-(2,6-dichloro-3-fluorophenyl)ethanone (1 g, 4.8 mmol) and 4-(benzyloxy)benzaldehyde (1.01 g, 4.8 mmol) in ethanol (10 ml), powdered KOH (0.40 g, 7.2 mmol) was added at 273 K. The reaction mixture was stirred at room temperature for 2 h. After completion of the reaction, the mixture was poured into ice cold water, acidified with HCl (1.5 N) until the pH value was approximately 3. The solid that precipitated was filtered and dried to afford the title compound as off-white solid, yield: 1.8 g (95%). Single crystals suitable for the X-ray diffraction study were grown from a mixture of toluene and acetone (*v*:*v* = 1:1) by slow evaporation at room temperature.

### Refinement

Carbon-bound H atoms were placed in calculated positions (C–H 0.95 Å for aromatic and vinylic carbon atoms, C–H 0.99 Å for methylene groups) and were included in the refinement in the riding model approximation, with *U*(H) set to 1.2*U*_eq_(C).

### Crystal data

**Table:**

C_22_H_15_Cl_2_FO_2_	*F*(000) = 824
*M**_r_* = 401.24	*D*_x_ = 1.399 Mg m^−^^3^
Monoclinic, *P*2_1_/*c*	Melting point = 369–367 K
Hall symbol: -P 2ybc	Mo *K*α radiation, λ = 0.71073 Å
*a* = 9.1977 (2) Å	Cell parameters from 8954 reflections
*b* = 21.6887 (4) Å	θ = 2.5–28.3°
*c* = 11.6072 (2) Å	µ = 0.36 mm^−^^1^
β = 124.629 (1)°	*T* = 200 K
*V* = 1905.29 (6) Å^3^	Cube, white
*Z* = 4	0.40 × 0.17 × 0.14 mm

### Data collection

**Table:**

Bruker APEXII CCD diffractometer	4712 independent reflections
Radiation source: fine-focus sealed tube	3729 reflections with *I* > 2σ(*I*)
Graphite monochromator	*R*_int_ = 0.027
φ and ω scans	θ_max_ = 28.3°, θ_min_ = 2.3°
Absorption correction: multi-scan (*SADABS*; Bruker, 2008)	*h* = −12→12
*T*_min_ = 0.681, *T*_max_ = 0.746	*k* = −20→28
17237 measured reflections	*l* = −15→15

### Refinement

**Table:**

Refinement on *F*^2^	Primary atom site location: structure-invariant direct methods
Least-squares matrix: full	Secondary atom site location: difference Fourier map
*R*[*F*^2^ > 2σ(*F*^2^)] = 0.041	Hydrogen site location: inferred from neighbouring sites
*wR*(*F*^2^) = 0.101	H-atom parameters constrained
*S* = 1.02	*w* = 1/[σ^2^(*F*_o_^2^) + (0.041*P*)^2^ + 0.8279*P*] where *P* = (*F*_o_^2^ + 2*F*_c_^2^)/3
4712 reflections	(Δ/σ)_max_ = 0.001
244 parameters	Δρ_max_ = 0.40 e Å^−^^3^
0 restraints	Δρ_min_ = −0.27 e Å^−^^3^

### Fractional atomic coordinates and isotropic or equivalent isotropic displacement parameters (Å^2^)

**Table:**

	*x*	*y*	*z*	*U*_iso_*/*U*_eq_	
Cl1	1.07669 (6)	0.33062 (2)	0.86095 (4)	0.05144 (14)	
Cl2	1.60001 (7)	0.17443 (2)	1.21644 (5)	0.05090 (14)	
F1	1.35724 (19)	0.38832 (5)	0.85238 (13)	0.0634 (3)	
O1	1.20670 (18)	0.23629 (6)	1.15009 (13)	0.0490 (3)	
O2	0.66727 (17)	−0.00811 (5)	0.41450 (12)	0.0422 (3)	
C1	1.2218 (2)	0.22034 (7)	1.05687 (16)	0.0338 (3)	
C2	1.1208 (2)	0.16972 (8)	0.96163 (17)	0.0384 (4)	
H2	1.0530	0.1445	0.9813	0.046*	
C3	1.1186 (2)	0.15692 (7)	0.84785 (16)	0.0319 (3)	
H3	1.1970	0.1800	0.8360	0.038*	
C4	0.6360 (2)	−0.00076 (8)	0.27948 (17)	0.0397 (4)	
H4A	0.7452	−0.0099	0.2848	0.048*	
H4B	0.6001	0.0422	0.2465	0.048*	
C11	1.3486 (2)	0.25530 (6)	1.03696 (14)	0.0283 (3)	
C12	1.2938 (2)	0.30701 (7)	0.95115 (15)	0.0323 (3)	
C13	1.4134 (3)	0.33938 (7)	0.93831 (17)	0.0394 (4)	
C14	1.5873 (3)	0.32290 (8)	1.01107 (18)	0.0421 (4)	
H14	1.6678	0.3463	1.0022	0.050*	
C15	1.6452 (2)	0.27204 (8)	1.09749 (17)	0.0393 (4)	
H15	1.7658	0.2601	1.1489	0.047*	
C16	1.5253 (2)	0.23868 (7)	1.10837 (15)	0.0316 (3)	
C21	1.0085 (2)	0.11127 (7)	0.73997 (16)	0.0316 (3)	
C22	0.8987 (2)	0.06930 (8)	0.74900 (17)	0.0397 (4)	
H22	0.9000	0.0682	0.8314	0.048*	
C23	0.7894 (3)	0.02991 (8)	0.64018 (18)	0.0423 (4)	
H23	0.7164	0.0018	0.6482	0.051*	
C24	0.7851 (2)	0.03105 (7)	0.51816 (16)	0.0343 (3)	
C25	0.8940 (2)	0.07133 (7)	0.50716 (17)	0.0340 (3)	
H25	0.8936	0.0719	0.4252	0.041*	
C26	1.0036 (2)	0.11089 (7)	0.61807 (17)	0.0338 (3)	
H26	1.0775	0.1386	0.6102	0.041*	
C31	0.4920 (2)	−0.04454 (7)	0.18024 (16)	0.0351 (3)	
C32	0.5074 (2)	−0.10705 (8)	0.21101 (17)	0.0392 (4)	
H32	0.6079	−0.1217	0.2969	0.047*	
C33	0.3791 (3)	−0.14809 (9)	0.11877 (18)	0.0459 (4)	
H33	0.3918	−0.1907	0.1414	0.055*	
C34	0.2328 (3)	−0.12764 (10)	−0.00587 (19)	0.0499 (5)	
H34	0.1447	−0.1561	−0.0695	0.060*	
C35	0.2144 (3)	−0.06611 (11)	−0.03803 (19)	0.0570 (5)	
H35	0.1131	−0.0519	−0.1240	0.068*	
C36	0.3439 (3)	−0.02427 (9)	0.05494 (19)	0.0489 (4)	
H36	0.3302	0.0183	0.0321	0.059*	

### Atomic displacement parameters (Å^2^)

**Table:**

	*U*^11^	*U*^22^	*U*^33^	*U*^12^	*U*^13^	*U*^23^
Cl1	0.0470 (3)	0.0639 (3)	0.0343 (2)	0.0235 (2)	0.01765 (19)	0.00794 (19)
Cl2	0.0530 (3)	0.0481 (3)	0.0527 (3)	0.0169 (2)	0.0307 (2)	0.0229 (2)
F1	0.0982 (10)	0.0362 (6)	0.0626 (7)	0.0085 (6)	0.0497 (7)	0.0160 (5)
O1	0.0587 (8)	0.0596 (8)	0.0430 (7)	−0.0113 (7)	0.0375 (7)	−0.0124 (6)
O2	0.0528 (7)	0.0349 (6)	0.0353 (6)	−0.0162 (5)	0.0228 (6)	−0.0089 (5)
C1	0.0340 (8)	0.0385 (8)	0.0293 (7)	−0.0024 (7)	0.0183 (6)	−0.0012 (6)
C2	0.0388 (9)	0.0404 (9)	0.0404 (8)	−0.0120 (7)	0.0251 (7)	−0.0061 (7)
C3	0.0299 (8)	0.0297 (7)	0.0336 (7)	−0.0026 (6)	0.0166 (6)	−0.0014 (6)
C4	0.0460 (9)	0.0328 (8)	0.0352 (8)	−0.0060 (7)	0.0200 (7)	−0.0020 (7)
C11	0.0337 (8)	0.0275 (7)	0.0219 (6)	−0.0026 (6)	0.0147 (6)	−0.0044 (5)
C12	0.0373 (8)	0.0321 (7)	0.0233 (7)	0.0029 (6)	0.0147 (6)	−0.0032 (6)
C13	0.0610 (11)	0.0252 (7)	0.0335 (8)	−0.0018 (7)	0.0277 (8)	0.0006 (6)
C14	0.0498 (10)	0.0384 (9)	0.0427 (9)	−0.0165 (8)	0.0291 (8)	−0.0073 (7)
C15	0.0339 (8)	0.0436 (9)	0.0362 (8)	−0.0060 (7)	0.0174 (7)	−0.0049 (7)
C16	0.0355 (8)	0.0292 (7)	0.0271 (7)	0.0012 (6)	0.0160 (6)	0.0017 (6)
C21	0.0304 (7)	0.0271 (7)	0.0334 (7)	−0.0014 (6)	0.0157 (6)	−0.0019 (6)
C22	0.0506 (10)	0.0338 (8)	0.0338 (8)	−0.0099 (7)	0.0235 (8)	−0.0017 (6)
C23	0.0532 (11)	0.0325 (8)	0.0405 (9)	−0.0151 (8)	0.0261 (8)	−0.0026 (7)
C24	0.0385 (9)	0.0235 (7)	0.0355 (8)	−0.0037 (6)	0.0178 (7)	−0.0043 (6)
C25	0.0351 (8)	0.0325 (8)	0.0365 (8)	−0.0018 (7)	0.0216 (7)	−0.0052 (6)
C26	0.0323 (8)	0.0316 (8)	0.0401 (8)	−0.0043 (6)	0.0221 (7)	−0.0051 (6)
C31	0.0375 (8)	0.0357 (8)	0.0298 (7)	−0.0025 (7)	0.0178 (7)	−0.0019 (6)
C32	0.0424 (9)	0.0380 (9)	0.0296 (8)	−0.0038 (7)	0.0160 (7)	0.0003 (6)
C33	0.0602 (12)	0.0400 (9)	0.0399 (9)	−0.0137 (9)	0.0299 (9)	−0.0075 (7)
C34	0.0494 (11)	0.0637 (12)	0.0352 (9)	−0.0186 (10)	0.0232 (8)	−0.0167 (8)
C35	0.0410 (10)	0.0772 (15)	0.0328 (9)	0.0045 (10)	0.0091 (8)	−0.0019 (9)
C36	0.0474 (11)	0.0455 (10)	0.0409 (9)	0.0067 (8)	0.0175 (8)	0.0052 (8)

### Geometric parameters (Å, º)

**Table:**

Cl1—C12	1.7230 (17)	C15—H15	0.9500
Cl2—C16	1.7344 (15)	C21—C26	1.389 (2)
F1—C13	1.3425 (18)	C21—C22	1.408 (2)
O1—C1	1.2167 (19)	C22—C23	1.376 (2)
O2—C24	1.3643 (18)	C22—H22	0.9500
O2—C4	1.4327 (19)	C23—C24	1.395 (2)
C1—C2	1.457 (2)	C23—H23	0.9500
C1—C11	1.515 (2)	C24—C25	1.388 (2)
C2—C3	1.338 (2)	C25—C26	1.391 (2)
C2—H2	0.9500	C25—H25	0.9500
C3—C21	1.460 (2)	C26—H26	0.9500
C3—H3	0.9500	C31—C36	1.383 (2)
C4—C31	1.500 (2)	C31—C32	1.389 (2)
C4—H4A	0.9900	C32—C33	1.377 (2)
C4—H4B	0.9900	C32—H32	0.9500
C11—C16	1.388 (2)	C33—C34	1.374 (3)
C11—C12	1.390 (2)	C33—H33	0.9500
C12—C13	1.383 (2)	C34—C35	1.370 (3)
C13—C14	1.366 (3)	C34—H34	0.9500
C14—C15	1.378 (2)	C35—C36	1.395 (3)
C14—H14	0.9500	C35—H35	0.9500
C15—C16	1.383 (2)	C36—H36	0.9500
			
C24—O2—C4	117.41 (12)	C26—C21—C3	118.88 (14)
O1—C1—C2	122.91 (15)	C22—C21—C3	123.48 (14)
O1—C1—C11	118.69 (14)	C23—C22—C21	120.93 (15)
C2—C1—C11	118.39 (13)	C23—C22—H22	119.5
C3—C2—C1	123.41 (15)	C21—C22—H22	119.5
C3—C2—H2	118.3	C22—C23—C24	120.38 (15)
C1—C2—H2	118.3	C22—C23—H23	119.8
C2—C3—C21	126.97 (15)	C24—C23—H23	119.8
C2—C3—H3	116.5	O2—C24—C25	124.31 (14)
C21—C3—H3	116.5	O2—C24—C23	115.75 (14)
O2—C4—C31	108.06 (13)	C25—C24—C23	119.92 (14)
O2—C4—H4A	110.1	C24—C25—C26	118.97 (14)
C31—C4—H4A	110.1	C24—C25—H25	120.5
O2—C4—H4B	110.1	C26—C25—H25	120.5
C31—C4—H4B	110.1	C21—C26—C25	122.23 (15)
H4A—C4—H4B	108.4	C21—C26—H26	118.9
C16—C11—C12	117.55 (14)	C25—C26—H26	118.9
C16—C11—C1	121.37 (13)	C36—C31—C32	118.46 (16)
C12—C11—C1	121.01 (14)	C36—C31—C4	121.24 (16)
C13—C12—C11	119.96 (15)	C32—C31—C4	120.27 (15)
C13—C12—Cl1	120.13 (12)	C33—C32—C31	120.94 (16)
C11—C12—Cl1	119.91 (13)	C33—C32—H32	119.5
F1—C13—C14	119.16 (16)	C31—C32—H32	119.5
F1—C13—C12	119.19 (17)	C34—C33—C32	120.26 (18)
C14—C13—C12	121.65 (15)	C34—C33—H33	119.9
C13—C14—C15	119.46 (16)	C32—C33—H33	119.9
C13—C14—H14	120.3	C35—C34—C33	119.79 (17)
C15—C14—H14	120.3	C35—C34—H34	120.1
C14—C15—C16	119.14 (16)	C33—C34—H34	120.1
C14—C15—H15	120.4	C34—C35—C36	120.27 (18)
C16—C15—H15	120.4	C34—C35—H35	119.9
C15—C16—C11	122.21 (14)	C36—C35—H35	119.9
C15—C16—Cl2	118.54 (13)	C31—C36—C35	120.29 (18)
C11—C16—Cl2	119.25 (12)	C31—C36—H36	119.9
C26—C21—C22	117.55 (14)	C35—C36—H36	119.9
			
O1—C1—C2—C3	170.87 (17)	C2—C3—C21—C26	169.97 (17)
C11—C1—C2—C3	−8.0 (2)	C2—C3—C21—C22	−6.4 (3)
C1—C2—C3—C21	−173.56 (16)	C26—C21—C22—C23	−0.6 (3)
C24—O2—C4—C31	−175.17 (14)	C3—C21—C22—C23	175.83 (16)
O1—C1—C11—C16	88.4 (2)	C21—C22—C23—C24	−0.2 (3)
C2—C1—C11—C16	−92.69 (18)	C4—O2—C24—C25	−9.4 (2)
O1—C1—C11—C12	−88.61 (19)	C4—O2—C24—C23	169.19 (15)
C2—C1—C11—C12	90.29 (18)	C22—C23—C24—O2	−177.49 (16)
C16—C11—C12—C13	0.7 (2)	C22—C23—C24—C25	1.2 (3)
C1—C11—C12—C13	177.82 (13)	O2—C24—C25—C26	177.34 (15)
C16—C11—C12—Cl1	−179.34 (11)	C23—C24—C25—C26	−1.2 (2)
C1—C11—C12—Cl1	−2.20 (19)	C22—C21—C26—C25	0.6 (2)
C11—C12—C13—F1	178.39 (13)	C3—C21—C26—C25	−176.04 (15)
Cl1—C12—C13—F1	−1.6 (2)	C24—C25—C26—C21	0.3 (2)
C11—C12—C13—C14	−1.7 (2)	O2—C4—C31—C36	127.10 (17)
Cl1—C12—C13—C14	178.27 (13)	O2—C4—C31—C32	−54.7 (2)
F1—C13—C14—C15	−178.82 (15)	C36—C31—C32—C33	0.4 (3)
C12—C13—C14—C15	1.3 (3)	C4—C31—C32—C33	−177.76 (16)
C13—C14—C15—C16	0.1 (2)	C31—C32—C33—C34	−0.1 (3)
C14—C15—C16—C11	−1.2 (2)	C32—C33—C34—C35	−0.3 (3)
C14—C15—C16—Cl2	179.46 (12)	C33—C34—C35—C36	0.3 (3)
C12—C11—C16—C15	0.8 (2)	C32—C31—C36—C35	−0.4 (3)
C1—C11—C16—C15	−176.37 (14)	C4—C31—C36—C35	177.73 (18)
C12—C11—C16—Cl2	−179.88 (11)	C34—C35—C36—C31	0.1 (3)
C1—C11—C16—Cl2	2.99 (19)		

### Hydrogen-bond geometry (Å, º)

*Cg* is the centroid of the C21–C26 ring.

**Table:**

*D*—H···*A*	*D*—H	H···*A*	*D*···*A*	*D*—H···*A*
C23—H23···F1^i^	0.95	2.55	3.375 (2)	145
C34—H34···Cl1^ii^	0.95	2.80	3.5462 (18)	136
C14—H14···*Cg*^iii^	0.95	2.51	3.297 (2)	140

Symmetry codes: (i) −*x*+2, *y*−1/2, −*z*+3/2; (ii) −*x*+1, *y*−1/2, −*z*+1/2; (iii) *x*+1, −*y*−1/2, *z*−1/2.
